# Visual Art Influences on Attitudes of Aging

**DOI:** 10.1093/geroni/igaf122.1679

**Published:** 2025-12-31

**Authors:** LaVona Traywick, Erica Fields

**Affiliations:** University of Oklahama, Oklahoma City, Oklahoma, United States; University of Arkansas Division of Agriculture, Little Rock, Arkansas, United States

## Abstract

The interplay between visual art and perceptions of aging is a powerful yet often overlooked avenue for fostering age-inclusive attitudes. Visual art influences both its creators and viewers, shaping societal narratives about aging. Historically, famous depictions of older adults in visual art have perpetuated stereotypes—some even subconscious—that reinforce negative perspectives on aging. These representations affect how society views older adults, often contributing to ageism. However, intentional shifts on how aging is portrayed can transform these perceptions. This presentation explores how visual art creation can be a catalyst to reshape the narrative of aging, by both professional artists and educational interventions for the public. The power of visual art to influence societal views of aging will be highlighted through showing historical and contemporary art examples. Additionally, a 4-H youth educational program will be shared that encompasses hands-on participation in visual art for the purpose of gerontological literacy. The youth reimagined their grandparents’ talents and skills as superpowers, cultivating a deeper appreciation for the unique contributions of older adults in their lives, as reflected through their artwork. Finally, the session will emphasize the critical role of gerontology professionals and educators in our everyday choice of imagery used in the classroom. Attendees will leave with a new awareness and actionable insights on how visual arts can promote, or hinder, positive perceptions of aging.

